# Trends in pediatric household cleaning product exposures before and during the COVID-19 pandemic: a national poison data system analysis (2016–2023)

**DOI:** 10.1186/s12887-026-07010-2

**Published:** 2026-05-21

**Authors:** Kayla J. Kendric, Timur S. Durrani

**Affiliations:** 1 California Poison Control System, University of California, Box 1369, San Francisco, CA 94143-1369 USA; 2 Division of Emergency Medicine, University of California, San Francisco, USA; 3 Division of Occupational and Environmental Medicine, University of California, San Francisco, San Francisco, USA

**Keywords:** Detergents, Pediatrics, Household products, COVID-19, Poison center

## Abstract

**Background:**

Unintentional exposures to household cleaning products remain a common cause of pediatric poison center calls in the United States, particularly among children under six. Most exposures involve toddlers and rarely result in severe outcomes. The impact of the COVID-19 pandemic on exposure patterns has not been well characterized. This study evaluates trends in pediatric exposures from 2016 to 2023, with a focus on changes in 2020.

**Methods:**

We conducted a retrospective analysis of unintentional exposures to household cleaning products reported to the National Poison Data System from 2016 to 2023 among children aged 0–5 years. Product types were grouped into 14 chemical categories. Descriptive statistics summarized exposure patterns, and interrupted time series analysis using segmented Poisson regression evaluated immediate and post-2020 changes in annual exposure trends associated with the COVID-19 pandemic.

**Results:**

A total of 633,317 unique exposures met inclusion criteria. Most involved children younger than 3 years (84.1%) and occurred at home (97.2%), with 87.7% managed on site. Significant clinical effects occurred in 2.7% of exposures with known outcomes. Soap and bleach products were the most frequent exposures, while alkali-based cleaners accounted for the highest number of moderate or more severe medical outcomes. Interrupted time series analysis demonstrated that several chemical classes experienced significant increases in exposures in 2020, most notably pine oil cleaners, bleach, soaps, and cationic cleaners.

**Conclusions:**

Pediatric exposures to household cleaning products remain common but showed an overall declining trend from 2016 to 2023, largely driven by decreases observed in alcohols/glycols, alkali, bleach, laundry, dishwasher, and soap products. Transient increases in exposures occurred during the COVID-19 pandemic, particularly for soaps, bleach, cationic cleaners, and pine oil products. Although severe outcomes were uncommon, alkali-based products posed the greatest risk.

**Supplementary Information:**

The online version contains supplementary material available at 10.1186/s12887-026-07010-2.

## Background

Unintentional poisonings remain a leading cause of injury among children in the United States, particularly those under six years of age. In 2024 alone, the America’s Poison Centers (APC) reported over 800,000 exposures in children aged five years or younger, with household cleaning substances accounting for 11.3% of all pediatric exposure calls, second only to cosmetics and personal care products [1]. Household cleaning products have been associated with both acute and chronic health effects in children, including respiratory irritation and exacerbation of pre-existing conditions [2–4].

Young children are at elevated risk for these exposures due to developmental behaviors such as mouthing, exploratory play, and limited understanding of hazard warnings. Household cleaning products, often stored in accessible locations and packaged in colorful containers, pose a particular threat. Prior studies have shown that most exposures involve toddlers aged 1–2 years, predominantly through ingestion, and commonly involve products such as bleach, disinfectants, and alkaline agents [5–8].

A 2023 analysis by Pacini et al. examined National Poison Data System (NPDS) data from 2000 to 2015 and found that while overall pediatric exposures to cleaning products declined during that period, most exposures involved toddlers, with ingestion as the primary route of exposure. Only 2.6% of exposures with known medical outcomes resulted in significant clinical effects or injury, but alkali-based products were disproportionately associated with severe outcomes, representing the highest number of exposures resulting in significant injury despite being the third most frequent exposure overall [5].

Although long-term trends suggest a decline in unintentional pediatric exposures, the COVID-19 pandemic may have disrupted this trajectory. Starting in March 2020, increased use of household cleaning and disinfectant products, as well as pandemic-related changes in childcare, school closures, remote work, and supervision, may have led to a temporary rise in pediatric exposures. Job loss and economic disruptions, particularly among workers in personal services, recreation, and tourism, may have further influenced childcare arrangements and household supervision. Prior reports demonstrated an increase in the daily poison center calls for exposures to cleaners and disinfectants in March 2020; however, no study has specifically evaluated the effect of the COVID-19 pandemic on cleaning product exposures in young children [9].

The present study builds on prior work by analyzing NPDS data from 2016 to 2023, focusing on trends before and after the onset of the COVID-19 pandemic [5]. We hypothesized that the incidence of pediatric exposures to household cleaning products reports to poison centers in the United States increased during the COVID-19 pandemic, particularly among high-use products such as soaps and bleach.

## Methods

We conducted a retrospective analysis using data from the America’s Poison Centers’ (APC) National Poison Data System (NPDS), a centralized database that collects near real-time information from all U.S. poison centers and includes standardized demographic, exposure, and clinical data.

Through a formal request to the APC, we obtained data on unintentional exposures to household cleaning products among children aged < 6 years from January 1, 2016, to December 31, 2023. All exposures classified as unintentional in NPDS were included, encompassing all unintentional exposure subcategories. Exposures were included if they involved one or more of the 83 NPDS generic product codes grouped under the major category “Cleaning Substances (Household),” as defined by the APC (Supplementary Material 1). Exposures were excluded if key variables, including age, gender, generic code number, exposure site, management site, or medical outcome, were missing. Follow-up calls performed by poison centers to monitor clinical progression did not affect inclusion; exclusions were based solely on missing key variables.

All exposures were included in the overall analysis. For analyses comparing exposures by specific product categories, only single-product exposures were retained, as multi-product exposures were excluded to allow clearer attribution of outcomes to a single product. Product exposures were grouped into 14 categories based on primary use, chemical class, and toxicologic mechanism (e.g., bleaches, alkali-based cleaners, soaps, disinfectants).

Medical outcomes were classified according to standard NPDS definitions as “no effect,” “minor effect,” “moderate effect,” “major effect,” and “death.” Minor effects involve minimally bothersome or transient symptoms, moderate effects involve more pronounced or prolonged symptoms requiring treatment, and major effects involve life-threatening or permanently disabling outcomes [10].

All data were de-identified, and the study was determined to be not human subjects research by the University of California, San Francisco Institutional Review Board, as investigators did not have access to identifiable information [11]. As such, informed consent to participate, including parental or legal guardian consent, was not required.

All exposures analyzed represent a population parameter. Reported outcomes per product category are presented as frequencies and apply only to this defined population. These values are descriptive and are not intended for inferential comparisons to broader populations, as NPDS data are based on voluntary reporting and lack a defined population denominator.

Descriptive statistics were used to summarize patient demographics, exposure characteristics, and clinical outcomes. Age was categorized into six groups: <1 year, 1–<2 years, 2–<3 years, 3–<4 years, 4–<5 years, and 5–<6 years. Gender was classified as male or female. Exposure site refers to the location where the exposure occurred and was categorized as own residence, other residence, school, public area, workplace, health care facility, restaurant/food service, or other. Caller site indicates the origin of the poison center call and was categorized similarly. Management site reflects the location or setting in which the patient was managed and was categorized as managed on site (non–health care facility), already in or en route to a health care facility at the time of the call, referred by the poison center to a health care facility, or other.

Annual exposure counts were calculated for each cleaning product category from 2016 to 2023. Interrupted time series (ITS) analysis using segmented Poisson regression was performed on aggregated annual call counts to evaluate changes associated with the onset of the COVID-19 pandemic. Regression coefficients were exponentiated to produce incidence rate ratios (IRRs), representing relative changes in expected call counts compared with the counterfactual trend. The model estimated both the immediate level change in 2020 and the post-2020 slope change relative to pre-pandemic trends. Counterfactual predictions were generated to estimate expected exposure counts had pre-2020 trends continued uninterrupted. ITS results were visualized using plots comparing observed counts with predicted counterfactual values. Formal estimation of effect size (e.g., excess calls) was not performed.

All statistical analyses were conducted in R (version 4.4.1; R Foundation for Statistical Computing) using RStudio (version 2024.09.0 + 375).

## Results

**Fig. 1:**
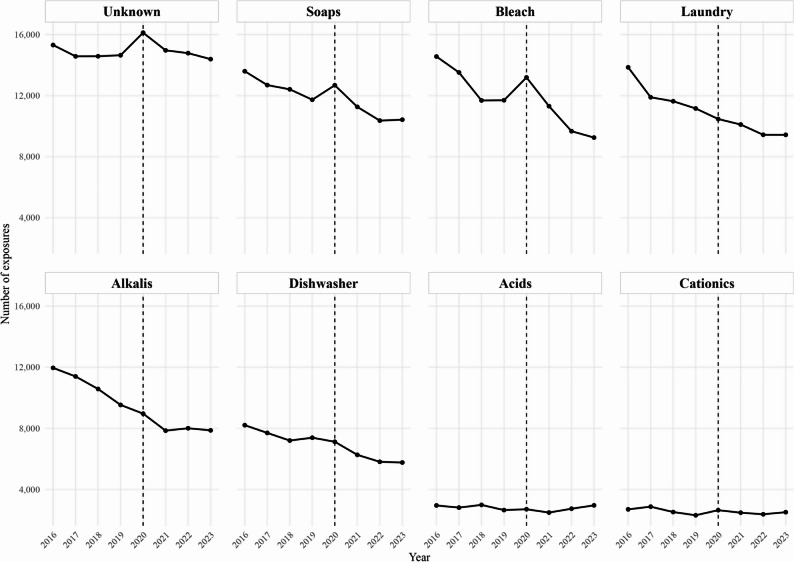
Annual trends in pediatric exposures to the eight most common household cleaning product categories reported to the National Poison Data System, 2016–2023. Annual pediatric household cleaning single-product exposures reported to NPDS from 2016 to 2023, shown as faceted line plots by chemical category. The eight categories with the highest cumulative exposures are displayed. The dashed vertical line denotes the onset of the COVID-19 pandemic in 2020

A total of 640,410 unique pediatric exposures were initially identified after collapsing multiple exposures per case to a single record. After excluding records with missing or “unknown” values in key variables, a total of 7,093 exposures were removed. Specifically, exclusions were made for gender (*n* = 1,135), exposure site (*n* = 1,803), caller site (*n* = 278), and management site (*n* = 3,877). No exposures were excluded for missing generic code, age, age unit, or medical outcome. For analyses by product category, only single-product exposures were retained, yielding a subset of 614,097 exposures (Supplemental Fig. 1). Patient demographics, exposure site, caller site, and management site are summarized in Table 1. Fig. 1 shows annual exposure trends for the eight household cleaning product categories with the highest cumulative exposure counts, while total annual exposure counts for all product categories, including both single- and multiple-product exposures, are provided in Supplemental Table 2.

**Table 1:** Demographic and exposure characteristics of pediatric household cleaning product exposures reported to U.S. Poison Centers, 2016–2023 (*n* = 633,317)

Demographic	*n*(%)
Age Group	
< 1 year	63,568 (10.0)
1–<2 years	282,940 (44.7)
2–<3 years	186,019 (29.4)
3–<4 years	60,132 (9.5)
4–<5 years	25,599 (4.0)
5–<6 years	15,059 (2.4)
Gender	
Female	267,335 (42.2)
Male	365,982 (57.8)
Exposure Site	
Own residence	615,623 (97.2)
Other residence	11,349 (1.8)
School	2374 (0.4)
Public area	1757 (0.3)
Workplace	272 (< 0.1)
Health care facility	180 (< 0.1)
Restaurant / food service	191 (< 0.1)
Other	1571 (0.2)
Caller Site	
Own residence	544,044 (85.9)
Health care facility	56,652 (8.9)
Other	19,190 (3.0)
Other residence	7955 (1.3)
Workplace	3054 (0.5)
Public area	1376 (0.2)
School	993 (0.2)
Restaurant / food service	53 (< 0.1)
Management Site	
Managed on site (non health care facility (HCF))	555,646 (87.7)
Patient already in (enroute to) HCF when poison control center (PCC) called	62,617 (9.9)
Patient was referred by PCC to a HCF	12,866 (2.0)
Other	2188 (0.3)
Medical Outcome	
No effect	139,642 (22.0)
Minor effect	92,831 (14.7)
Moderate effect	6332 (1.0)
Major effect	384 (0.1)
Death	6 (< 0.1)
Confirmed non-exposure	281 (< 0.1)
Not followed, judged as nontoxic exposure	44,303 (7.0)
Not followed, minimal clinical effects possible	335,531 (53.0)
Unable to follow, judged as a potentially toxic exposure	6654 (1.1)
Unrelated effect, the exposure was probably not responsible for the effect(s)	7353 (1.2)

Data are presented as counts (*n*) and percentages (%). Percentages are calculated using available data for each variable, excluding cases with missing or unknown values. Exposure site refers to the location where the exposure occurred. Caller site indicates the origin of the call to the poison control center. Management site reflects where the patient was managed, including non health care facility (HCF) and HCF settings

*PCC* Poison control center, *HCF* Health care facility

The majority of exposures were classified as “Unintentional - General” exposures (*n* = 624,127, 98.5%). Among exposures with known age and gender, the majority (84.1%) involved children younger than 3 years old, with a peak at 1–<2 years of age (44.7%) (Table 1). Males accounted for 57.8% of exposures (Table 1). Most patients (87.7%) were managed on site (non-health care facility), with only 2.0% referred to a health care facility by poison centers (Table 1). The proportion of exposures occurring in the home remained stable across the study period, accounting for approximately 97% of cases in the pre-pandemic (97.0%), pandemic (97.7%), and post-pandemic (97.3%) periods. Of single-substance exposures with known medical outcomes (*n* = 231,111; 37.6% of total single-substance exposures), no effect was the most reported outcome (*n* = 135,300; 58.5%), followed by minor effect (*n* = 89,285; 38.6%), moderate effect (*n* = 5,919; 2.6%), major effect (*n* = 334; 0.1%), and death (*n* = 5; <0.01%) (Table 2). Serious medical outcomes (defined as moderate, major, or death outcomes) accounted for 2.7% of exposures with known outcomes. The distribution of exposures by household cleaning product category and associated medical outcomes is presented in Table 2.

**Table 2:** Medical outcomes of pediatric household cleaning single-product exposures by chemical class, 2016–2023 (*n* = 614,097)

Chemical Class	Total exposures, *n* (% of all exposures)	Exposures with known medical outcome, *n* (% of all exposures with known medical outcome)	Death	Major	Moderate	Minor	No Effect	Confirmed non-exposure	Total moderate or above medical outcome; *n* (% of exposures with known medical outcome per class)
Acids	22,332 (3.6%)	10,074 (4.4%)	1	38	293	3163	6561	18	332 (3.3%)
Alcohols/Glycols	19,565 (3.2%)	6326 (2.7%)	0	3	69	1651	4595	8	72 (1.1%)
Alkali	76,166 (12.4%)	33,984 (14.7%)	0	129	1471	12,811	19,529	44	1600 (4.7%)
Ammonia	6104 (1.0%)	2174 (0.9%)	0	2	22	582	1565	3	24 (1.1%)
Bleach	94,869 (15.5%)	43,174 (18.7%)	0	26	1057	21,806	20,252	33	1083 (2.5%)
Borates	2087 (0.3%)	932 (0.4%)	0	0	29	404	497	2	29 (3.1%)
Cationic	20,451 (3.3%)	7427 (3.2%)	0	6	190	2961	4262	8	196 (2.6%)
Hydrofluoric Acid	211 (0.0%)	168 (0.1%)	1	1	13	66	87	0	15 (8.9%)
Dishwasher	55,484 (9.0%)	18,982 (8.2%)	0	6	281	7321	11,333	41	287 (1.5%)
Laundry	87,962 (14.3%)	30,759 (13.3%)	0	32	867	12,590	17,242	28	899 (2.9%)
Phenol	1731 (0.3%)	669 (0.3%)	0	0	7	219	443	0	7 (1.0%)
Pine Oil	11,664 (1.9%)	4835 (2.1%)	0	3	122	1580	3126	4	125 (2.6%)
Soap	95,150 (15.5%)	30,876 (13.4%)	3	39	768	12,010	18,032	24	810 (2.6%)
Starches	783 (0.1%)	227 (0.1%)	0	0	2	37	187	1	2 (0.9%)
Other	164 (0.0%)	50 (0.0%)	0	0	0	8	42	0	0 (0.0%)
Unknown	119,374 (19.4%)	40,454 (17.5%)	0	49	728	12,076	27,547	54	777 (1.9%)
Total	614,097	231,111	5	334	5919	89,285	135,300	268	6258 (2.7%)

Data are presented as counts (n) and percentages (%). Medical outcomes are categorized as no effect, minor effect, moderate effect, major effect, death, and confirmed non-exposure, according to National Poison Data System (NPDS) definitions*. “Moderate or greater outcomes” include moderate effect, major effect, and death, and are reported as a percentage of exposures with known medical outcomes for each chemical class. The number of exposures with known medical outcomes and the total number of exposures are provided for each chemical class; percentages of total exposures are calculated relative to all reported exposures across the study period. Percentages represent column percentages for columns “Total exposures, n (% of all exposures)” and “Exposures with known medical outcome, n (% of all exposures with known medical outcome)” and row percentages for column “Total moderate or above medical outcome; n (% of exposures with known medical outcome per class)”. Percentages are based on available data and may not sum to 100% due to rounding

*Medical outcome definitions according to NPDS: Minor = minimally bothersome/transient symptoms; Moderate = pronounced/prolonged symptoms requiring treatment; Major = life-threatening or permanently disabling outcome

A sensitivity analysis was conducted including cases involving multiple-substance exposures (*n* = 19,220; 3.0% of total exposures) (Supplemental Table 3). These exposures included cases involving more than one cleaning product and, in some instances, co-exposures to non-cleaning substances such as medications or other non-pharmaceutical agents. Multiple-substance exposures accounted for a small proportion of total cases and demonstrated a similar overall distribution of medical outcomes compared with single-substance exposures. However, a statistically significant difference in outcome distribution was observed (χ² = 369.4, df = 4, *p* < 0.001), driven by a modest increase in moderate and major outcomes among multiple-substance exposures.

The five fatalities to single-product exposures included one acidic cleaning product, one hydrofluoric cleaning product, and three soap exposures. Although soap-based products are generally associated with low-severity outcomes, the three fatalities attributed to soap exposures all involved children aged < 2 years. These exposures were classified under the generic category of wall, floor, tile, or all-purpose cleaning agents containing anionic or nonionic surfactants. All three patients required admission to a critical care unit and were already en route to or present at a health care facility at the time of poison center contact. The additional fatality identified in the multiple-substance cohort involved a 14-month-old male with co-exposure to a cationic cleaning agent and a non-prescription fentanyl product; the exposure occurred at home, and the patient was admitted to a critical care unit and subsequently died (Supplemental Table 3).

By chemical class, soap products were the most frequently implicated (*n* = 95,150; 15.49%), followed closely by bleach products (*n* = 94,869; 15.45%) (Supplemental Table 2). Among alkali exposures with known medical outcomes (*n* = 33,984), 4.7% (*n* = 1,600) resulted in moderate, major, or fatal clinical outcomes, representing the highest number of significant outcomes among all product categories, despite alkali products being the fourth most frequent exposure (Table 2). Alkali exposures were associated with 129 major outcomes, the highest of any chemical class. The proportion of moderate or greater medical outcomes in the alkali category was exceeded only by hydrofluoric acid, a far less common exposure.

The alkali category was delineated into seven specific generic codes (Table 3). The most common exposures were to miscellaneous alkali-based cleaning agents (33.8% of all alkali exposures; *n* = 25,735) and to all-purpose alkali cleaners, including wall, floor, and tile cleaners, which comprised 33.7% (*n* = 25,684). Although these two subcategories accounted for the majority of alkali exposures, they were less frequently associated with moderate or major medical outcomes. In contrast, alkali-based oven cleaners (*n* = 282) and drain cleaners (*n* = 297) had the highest proportions of moderate or greater medical outcomes (20.1% and 20.9%, respectively), despite representing only 2.8% and 3.0% of all alkali exposures. These findings are consistent with those reported by Pacini et al., which similarly identified oven and drain cleaners as leading sources of severe alkali-related injuries despite their lower overall exposure frequency [5].

**Table 3:** Medical outcomes of pediatric alkali-based cleaning product exposures by generic code category, 2016–2023 (*n* = 76,166)

Generic Code Name	Total exposures, *n* (% of all alkali exposures)	Exposures with known medical outcome, *n* (% of all exposures with known medical outcome)	Major	Moderate	Minor	No Effect	Confirmed non-exposure	Total moderate or above medical outcome; *n* (% of exposures with known medical outcome per sub generic code name)
Drain Cleaners	2254 (3.0%)	1475 (4.3%)	44	253	559	614	5	297 (20.1%)
Industrial Cleaners	2757 (3.6%)	1422 (4.2%)	16	200	569	635	2	216 (15.2%)
Misc Cleaning agents	25,735 (33.8%)	10,597 (31.2%)	34	336	3968	6242	17	370 (3.5%)
Oven Cleaners	2141 (2.8%)	1347 (4.0%)	21	261	725	337	3	282 (20.9%)
Rust Removers	7 (0.0%)	4 (0.0%)	0	2	1	1	0	2 (50.0%)
Toilet Bowl Cleaners	17,588 (23.1%)	7459 (21.9%)	2	89	1833	5528	7	91 (1.2%)
Wall/Floor/Tile/All-Purpose Cleaning Agents	25,684 (33.7%)	11,680 (34.4%)	12	330	5156	6172	10	342 (2.9%)
Total	76,166	33,984	129	1471	12,811	19,529	44	1600 (4.7%)

Data are presented as counts (n) and percentages (%). Alkali-based cleaning product exposures are stratified by NPDS generic code categories. Total exposures for each category are reported as a proportion of all alkali exposures. Medical outcomes are categorized as no effect, minor effect, moderate effect, major effect, and confirmed non-exposure, according to National Poison Data System (NPDS) definitions. Percentages for individual outcome categories are calculated using the number of exposures with known medical outcomes within each alkali category. “Moderate or above outcomes” include moderate effect and major effect and are reported as a percentage of exposures with known medical outcomes for each category. Percentages represent column percentages for columns “Total exposures, n (% of all alkali exposures)” and “Exposures with known medical outcome, n (% of all exposures with known medical outcome)” and row percentages for column “Total moderate or above medical outcome; n (% of exposures with known medical outcome per sub generic code name)”. Percentages are based on available data and may not sum to 100% due to rounding

Interrupted time series (ITS) analysis demonstrated that several chemical classes experienced statistically significant immediate increases in exposures in 2020 above expected pre-pandemic trends (Table 4). Relative changes in annual call counts are reported as incidence rate ratios (IRRs) from the segmented Poisson regression model. Pine oil cleaners exhibited the largest relative spike (IRR 1.49, 95% CI 1.38–1.61, *p* < 0.001), followed by bleach (IRR 1.29, 95% CI 1.26–1.33, *p* < 0.001), unknown cleaning products (IRR 1.14, 95% CI 1.11–1.16, *p* < 0.001), soaps (IRR 1.13, 95% CI 1.10–1.16, *p* < 0.001), cationic cleaners (IRR 1.10, 95% CI 1.04–1.17, p = < 0.001), and dishwasher detergents (IRR 1.04, 95% CI 1.00–1.08, *p* = 0.038).

**Table 4:** Interrupted time series regression analysis of changes in pediatric household cleaning product exposures associated with the onset of the COVID-19 pandemic, NPDS, 2016–2023

Category	2020 Level Change (Immediate Spike) - IRR	2020 Level Change (Immediate Spike) − 95% CI	2020 Level Change (Immediate Spike) - *p*-value	Post-2020 Slope Change - IRR	Post-2020 Slope Change − 95% CI	Post-2020 Slope Change - *p*-value
Acids	0.9	0.85–0.96	< 0.001	1.06	1.04–1.09	< 0.001
Alcohols/Glycols	0.91	0.85–0.97	0.004	1.09	1.06–1.12	< 0.001
Alkalis	0.93	0.9–0.96	< 0.001	1.04	1.02–1.05	< 0.001
Ammonia	0.76	0.67–0.85	< 0.001	0.95	0.9–1	0.042
Bleach	1.29	1.26–1.33	< 0.001	0.96	0.95–0.97	< 0.001
Borates	0.22	0.18–0.28	< 0.001	0.49	0.45–0.55	< 0.001
Cationic	1.1	1.04–1.17	< 0.001	1.04	1.01–1.06	0.002
Dishwasher	1.04	1–1.08	0.038	0.97	0.95–0.98	< 0.001
Hydrofluoric Acid	1.18	0.65–2.14	0.581	0.98	0.76–1.26	0.876
Laundry	0.99	0.97–1.02	0.661	1.03	1.02–1.04	< 0.001
Other	0.73	0.37–1.45	0.369	1.18	0.89–1.55	0.246
Phenol	0.91	0.72–1.15	0.414	1.45	1.32–1.59	< 0.001
Pine oil	1.49	1.38–1.61	< 0.001	0.98	0.95–1.01	0.23
Soaps	1.13	1.1–1.16	< 0.001	0.98	0.97–0.99	< 0.001
Starches	0.63	0.44–0.89	0.009	1	0.87–1.16	0.95
Unknown	1.14	1.11–1.16	< 0.001	0.98	0.97–0.99	< 0.001

Interrupted time series regression evaluating changes in pediatric household cleaning product exposures following the onset of the COVID-19 pandemic in 2020. The 2020 level change reflects the immediate change in exposure rates, and the post-2020 slope change reflects the change in monthly trend compared to the pre-pandemic period. Results are presented as incidence rate ratios (IRRs) with 95% confidence intervals (CIs) and *p*-values. IRR > 1 indicates an increase in exposures; IRR < 1 indicates a decrease. A two-sided *p*-value < 0.05 was considered statistically significant

Several categories showed significant decreases in 2020, including ammonia (IRR 0.76, 95% CI 0.67–0.85, *p* < 0.001), alkalis (IRR 0.93, 95% CI 0.90–0.96, *p* < 0.001), alcohols/glycols (IRR 0.91, 95% CI 0.85–0.97, *p* = 0.004), and starches (IRR 0.63, 95% CI 0.44–0.89, *p* = 0.009) (Table 4).

Post-2020 trends differed by chemical class. Exposures to acids, alcohols/glycols, alkalis, cationic cleaners, laundry products, and phenol-based cleaners all showed significant positive post-2020 slope changes, indicating continued increases after 2020 (IRR range 1.03–1.45, all *p* ≤ 0.01). In contrast, ammonia, bleach, borates, dishwasher products, soaps, and unknown cleaning products demonstrated significant negative post-2020 slope changes (IRR range 0.49–0.98, all *p* ≤ 0.05), consistent with gradual declines in exposures following the initial pandemic period (Table 4).

**Fig. 2:**
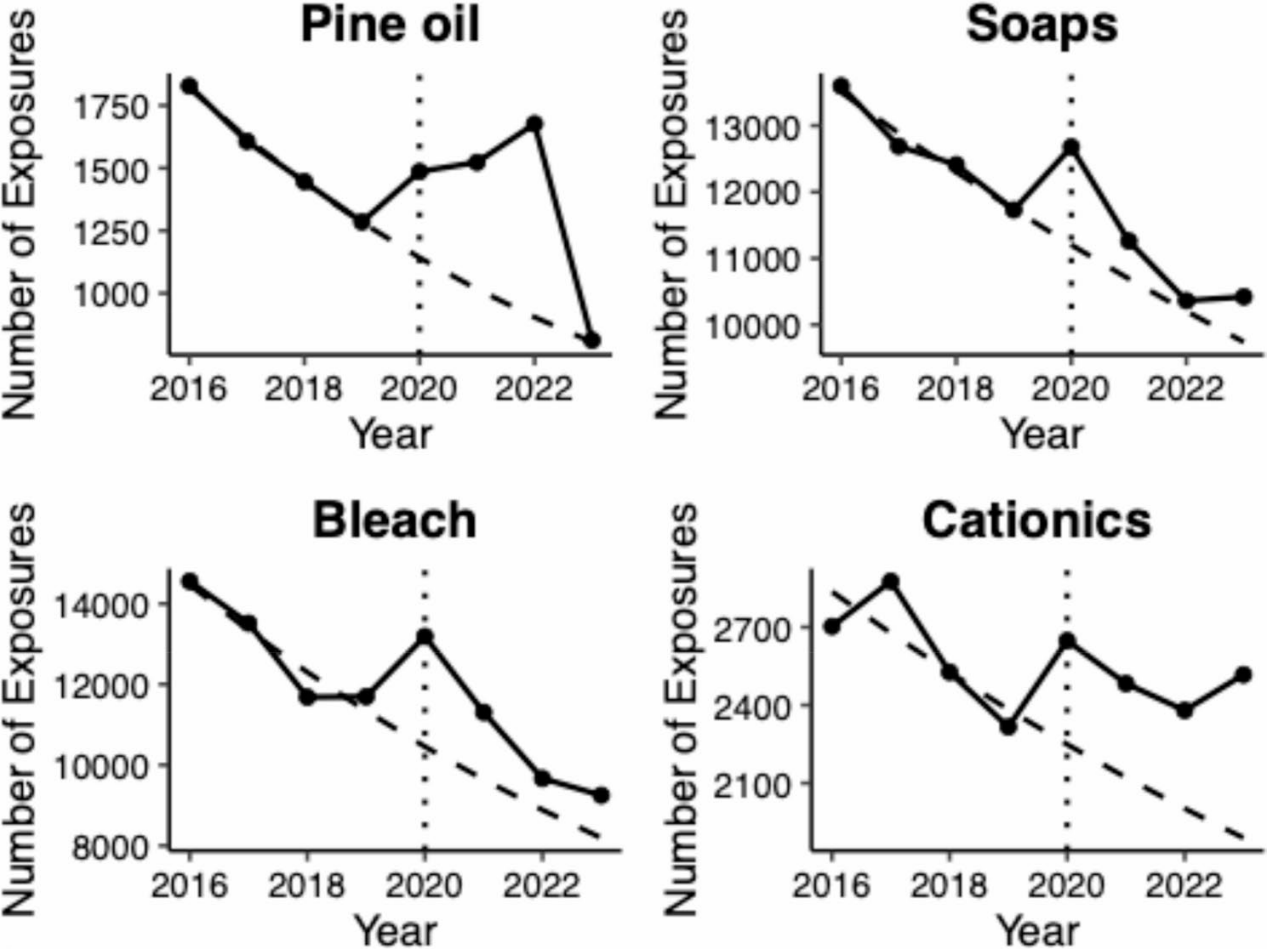
Interrupted time series analysis of pediatric exposures to pine oil, soaps, bleach, and cationic cleaning agents before and during the COVID-19 pandemic, NPDS, 2016–2023. Solid lines represent observed exposure counts, and dashed lines represent counterfactual predictions estimating expected exposures had pre-pandemic trends continued. The dotted vertical line marks the onset of the COVID-19 pandemic in 2020

ITS plots for selected chemical classes (pine oil, soaps, bleach, and cationic cleaners) are shown in Fig. 2. All four classes deviated from expected counterfactual trends in 2020, with observed exposures exceeding predicted values based on pre-pandemic trajectories. Pine oil exhibited the most pronounced pattern, with exposures declining before 2020, followed by a sharp and statistically significant spike in 2020 and continued elevated activity through 2022 before a marked decrease in 2023. Soaps and bleach similarly showed immediate increases in 2020 with subsequent gradual declines, while cationic cleaners displayed a smaller but noticeable 2020 elevation with more stable trends thereafter.

## Discussion

Our analysis demonstrates that pediatric exposures to household cleaning products increased during the COVID-19 pandemic, particularly for high-use products such as soaps and bleach, supporting our original hypothesis. Most exposures occurred in children aged 0–<3 years and primarily took place in the home, consistent with prior literature [5–8]. The majority of exposures were classified as unintentional-general exposures, further supporting exploratory ingestion as the primary mechanism of exposure in this age group. While several chemical classes experienced short-term spikes in 2020, overall exposures across the 2016–2023 period were stable or declined for many product types.

The distribution of exposures by chemical class largely mirrors prior findings, with one notable difference: laundry agents accounted for 14.3% of exposures in the present dataset, compared with 0.5% in earlier reports [5]. This difference may reflect variations in study years, product coding, or a true increase in exposures to laundry detergent packets, which have become more prevalent in households over the past decade [12, 13]. Although product-level data limited our ability to fully distinguish packet from non-packet detergents, the continued association between laundry products and higher-severity outcomes supports ongoing concern regarding concentrated formulations and visually appealing yet insufficient child-resistant packaging.

Interrupted time series (ITS) analysis revealed significant immediate increases in 2020 across multiple chemical classes, including acids, soaps, bleach, cationic cleaners, and pine oil, relative to counterfactual trends. These findings align with prior reports of increased household cleaning and disinfection behaviors early in the COVID-19 pandemic [12–14]. For most categories, exposures subsequently declined toward baseline in the years following 2020, consistent with the relaxation of shelter-in-place orders and a gradual return to pre-pandemic routines as children resumed daycare and school attendance. In contrast, several product categories, including alcohols/glycols, ammonia, laundry agents, and dishwasher detergents, demonstrated stable or downward trends during the pandemic period, suggesting that shifts in household product use and associated exposures were not uniform across cleaning product types. Pine oil cleaners exhibited a distinct pattern, with exposures continuing to rise through 2022 before declining sharply in 2023. This trajectory may reflect increased post-pandemic use of pine oil–based products for perceived “natural” or disinfecting properties, seasonal cleaning behaviors, or household practices that differ from other chemical classes. Pine oil cleaners are often marketed for multi-surface use and may be more accessible in homes with young children, potentially increasing exposure risk. Further research is warranted to determine whether product-specific characteristics, marketing trends, or behavioral patterns contributed to this unique exposure trajectory.

Our findings are consistent with both U.S. and international reports demonstrating increased exposures to cleaning products during the COVID-19 pandemic. Chang et al. reported increased U.S. poison center calls for cleaning agents and disinfectants in early 2020, while Canadian and French data similarly showed elevated exposure calls during national lockdown periods [9, 15, 16]. Differences in the magnitude and duration of these trends likely reflect variation in public health messaging, lockdown measures, and household cleaning practices across regions.

Although total pediatric exposure calls increased from 2019 to 2020, calls declined in 2021 and generally trended downward across the study period (Supplemental Table 2). This decline may reflect improved caregiver education on safe storage and use, adoption of child-resistant packaging, broader public health initiatives, and changes in poison center utilization, such as increased use of online resources or telehealth. Continued surveillance is warranted to monitor whether these declines persist and to identify ongoing opportunities for prevention. Although the COVID-19 pandemic represents a unique historical event, the observed exposure surges highlight how rapid changes in household behavior, product availability, and cleaning practices can meaningfully alter pediatric poisoning risk. For clinicians and poison centers, these findings support heightened vigilance during periods of societal disruption, supply shortages, or public health messaging that encourage increased disinfectant use. Identifying product categories associated with both higher exposure frequency and greater clinical severity may inform anticipatory guidance, triage decisions, and targeted prevention efforts during future public health emergencies.

Across all study years, only 2.7% of exposures with known medical outcomes resulted in significant clinical effects, underscoring that most exposures were low risk. Nevertheless, certain product classes consistently posed greater hazard. Alkali-based cleaning products accounted for the highest number of exposures with clinically significant outcomes, reinforcing longstanding concerns regarding their corrosive potential and the need for targeted prevention efforts [5].

Although soap-based products are generally low-risk, rare fatalities in this cohort highlight that even commonly used household cleaners can cause severe outcomes in vulnerable populations, particularly children under 2 years old. All three soap-related fatalities involved wall, floor, tile, or all-purpose cleaning agents containing anionic or nonionic surfactants. Each patient required critical care admission and was already en route to or present at a health care facility at the time of poison center contact. The additional fatality identified in the multiple-substance cohort involved co-exposure to a cationic cleaning agent and a non-prescription fentanyl product, limiting the ability to attribute toxicity to a single agent and highlighting the complexity of multi-substance exposures. Given NPDS limitations, including lack of information on dose, formulation, and contributing clinical factors, these deaths may reflect aspiration, co-exposures, or misclassification rather than toxicity from surfactants alone. Nonetheless, these rare events underscore that severe outcomes can occur even with products perceived as benign. Taken together, these findings highlight ongoing opportunities for public health interventions focused on safe storage and handling of cleaning products, particularly in households with young children. Enhanced caregiver education, improved child-resistant packaging, and regulatory strategies directed at higher-risk agents such as alkali cleaners and laundry packets may help mitigate future unintentional pediatric exposures.

This study has several limitations. As an observational analysis of poison center data, causal relationships between exposures and external influences, including pandemic-related behavioral changes, cannot be definitively established. Analyses comparing product categories were restricted to single-product exposures to allow clearer attribution of outcomes and to reduce potential misclassification that may occur when multiple product categories are involved in a single exposure. Multi-product exposures may include combinations of cleaning agents or co-exposures to substances outside the household cleaning product category (e.g., medications, health/beauty products, essential oils, illicit substances), introducing additional complexity in interpretation. These excluded exposures may differ in clinical severity or management and represent an area for future research.

An analysis including multiple-substance exposures was conducted to assess the robustness of the primary findings (Supplemental Table 3). Although this analysis demonstrated a statistically significant difference in medical outcome distribution compared with single-substance exposures, the absolute differences were small, and the overall pattern of outcomes remained similar. These findings suggest that exclusion of multiple-substance exposures did not meaningfully alter study conclusions. Misclassification of product categories or medical outcomes remains possible, and coding practices may vary across specialists and over time [1].

Poison center data likely underestimate true exposure incidence because reporting is voluntary and contingent on caregiver recognition, access, and willingness to seek guidance [1]. Most exposures were self-reported and could not be independently verified, introducing the potential for inaccuracies in exposure details, product identification, and symptom reporting. Because NPDS data lack a defined population denominator, findings cannot be generalized directly to the broader population. Future studies incorporating population-based denominators or linkage to external datasets may allow for more robust inference regarding incidence and risk.

Our dataset lacks information on parental employment, childcare arrangements, or remote work status; limiting assessment of these factors in exposure patterns. Geographic information was unavailable, restricting evaluation of spatial differences in region-specific pandemic effects. Incomplete medical outcome data and the small proportion of documented severe exposures further limit conclusions regarding clinical severity.

## Conclusions

Unintentional pediatric exposures to household cleaning products remained common throughout the study period, although overall call volumes demonstrated a gradual decline before and after the COVID-19 pandemic, suggesting that ongoing public health efforts have been effective. Despite this broader downward trend, several chemical classes, including soaps, bleach, cationic cleaners, and pine oil products, experienced significant spikes in 2020 during the pandemic. Ingestion was found to be the most frequent route of exposure. While most exposures resulted in minimal clinical effects, certain agents, particularly alkali cleaners, continued to pose disproportionate risk. These findings highlight the need for ongoing prevention strategies focused on safe storage, caregiver education, and targeted regulatory efforts, especially during periods of societal disruption or heightened product use.

## Supplementary Information

Supplementary Material 1.

Supplementary Material 2: Supplemental Table 1. APC Cleaning Substances (Household) Generic Code Name and Number. Supplemental Figure 1. Flow diagram of pediatric household cleaning product exposures included in the analysis. Starting with 640,410 unique reported exposures, records with missing, blank, or “unknown” values in key variables (Generic Code, Age, Age Unit, Gender, Exposure Site, Caller Site, Management Site, or Medical Outcome) were sequentially excluded, leaving 633,317 exposures for the main analyses. Subsequent analyses by single product category excluded exposures involving multiple products, resulting in 614,097 exposures included in these comparisons. Supplemental Table 2. Household cleaning product exposure prevalence by year for all pediatric exposures, NPDS, 2016–2023 (n = 633,317). Supplemental Table 3. Medical outcomes of pediatric household cleaning product exposures stratified by single- and multiple-substance exposures, 2016–2023.

## Data Availability

The data that support the findings of this study are available from the corresponding author, Dr. Kayla Kendric, upon reasonable request.

## Abbreviations

APC: America’s Poison Centers
NPDS: National Poison Data System
COVID-19: Coronavirus Disease 2019
ITS: Interrupted time series
IRR: Incidence Rate Ratio
CI: Confidence Interval
HCF: Health Care Facility
PCC: Poison Control Center

## References

[1] Beuhler MC, Feldman R, Gummin DD, et al. 2024 Annual report of the National Poison Data System^®^ (NPDS) from America’s Poison Centers^®^: 42nd annual report. Clin Toxicol. 2025;63(12):1029–280. 10.1080/15563650.2025.2571299. 10.1080/15563650.2025.2571299 41432769

[2] Parks J, McCandless L, Dharma C, et al. Association of use of cleaning products with respiratory health in a Canadian birth cohort. Can Med Assoc J. 2020;192(7):E154–61. 10.1503/cmaj.190819. 32071106 10.1503/cmaj.190819 PMC7030878

[3] Weinmann T, Gerlich J, Heinrich S, et al. Association of household cleaning agents and disinfectants with asthma in young German adults. Occup Environ Med. 2017;74(9):684–90. 10.1136/oemed-2016-104086. 28483971 10.1136/oemed-2016-104086

[4] Nickmilder M, Carbonnelle S, Bernard A. House cleaning with chlorine bleach and the risks of allergic and respiratory diseases in children. Pediatr Allergy Immunol. 2007;18(1):27–35. 10.1111/j.1399-3038.2006.00487.x. 17295796 10.1111/j.1399-3038.2006.00487.x

[5] Pacini A, Tsutaoka B, Lai L, Durrani TS. Unintentional pediatric exposures to household cleaning products: a cross-sectional analysis of the National Poison Data System (2000–2015). J Occup Med Toxicol Lond Engl. 2023;18(1):16. 10.1186/s12995-023-00384-4. 10.1186/s12995-023-00384-4 PMC10422824 37568177

[6] Yen CW, Lee EP, Cheng SC, Hsia SH, Huang JL, Lee J. Household cleaning products poisoning in a pediatric emergency center: A 10- year cross-sectional study and literature review. Pediatr Neonatol. 2021;62(6):638–46. 10.1016/j.pedneo.2021.05.026. 34332912 10.1016/j.pedneo.2021.05.026

[7] McKenzie LB, Ahir N, Stolz U, Nelson NG. Household Cleaning Product-Related Injuries Treated in US Emergency Departments in 1990–2006. Pediatrics. 2010;126(3):509–16. 10.1542/peds.2009-3392. 20679298 10.1542/peds.2009-3392

[8] Franklin RL, Rodgers GB. Unintentional Child Poisonings Treated in United States Hospital Emergency Departments: National Estimates of Incident Cases, Population-Based Poisoning Rates, and Product Involvement. Pediatrics. 2008;122(6):1244–51. 10.1542/peds.2007-3551. 19047241 10.1542/peds.2007-3551

[9] Chang A. Cleaning and Disinfectant Chemical Exposures and Temporal Associations with COVID-19 — National Poison Data System, United States, January 1, 2020–March 31, 2020 10.15585/mmwr.mm6916e1. 10.15585/mmwr.mm6916e1 PMC7188411 32324720

[10] NPDS Coding User Manual. June. pdf.https://www.npds.us/Help/NPDS%20Coding%20User%20Manual%20(June%202023).pdf. Accessed 29 May 2025.

[11] UCSF IRB Not Human Subjects Research. Human Research Protection Program (HRPP). 2025. https://irb.ucsf.edu/not-human-subjects-research. Accessed 17 Mar 2026.

[12] Zhang AM, Smith GA, Casavant MJ, Kistamgari S, Gaw CE. Longitudinal trends in liquid laundry detergent packet exposures: 2014–2022. Clin Toxicol. 2023;61(11):990–8. 10.1080/15563650.2023.2287977. 10.1080/15563650.2023.2287977 38112310

[13] Valdez AL, Casavant MJ, Spiller HA, Chounthirath T, Xiang H, Smith GA. Pediatric exposure to laundry detergent pods. Pediatrics. 2014;134(6):1127–35. 10.1542/peds.2014-0057. 25384489 10.1542/peds.2014-0057

[14] Davis MG, Casavant MJ, Spiller HA, Chounthirath T, Smith GA. Pediatric Exposures to Laundry and Dishwasher Detergents in the United States: 2013–2014. Pediatrics. 2016;137(5). 10.1542/peds.2015-4529. 10.1542/peds.2015-4529 27244825

[15] Yasseen Iii A, Weiss D, Remer S, et al. Increases in exposure calls related to selected cleaners and disinfectants at the onset of the COVID-19 pandemic: data from Canadian poison centres. Health Promot Chronic Dis Prev Can. 2021;1(1):25–9. 10.24095/hpcdp.41.1.03. 10.24095/hpcdp.41.1.03 PMC7852621 33438943

[16] Le Roux G, Sinno-Tellier S, Descatha A. COVID-19: home poisoning throughout the containment period. Lancet Public Health. 2020;5(6):e314. 10.1016/S2468-2667(20)30095-5. 32339480 10.1016/S2468-2667(20)30095-5 PMC7182512

