# Supplementary material for: Trends in pediatric household cleaning product exposures before and during the COVID-19 pandemic: a national poison data system analysis (2016–2023)
Source: BMC Pediatr. 2026 May 21;26:658. doi: 10.1186/s12887-026-07010-2 (PMC13371303; doi:10.1186/s12887-026-07010-2)
Supplement: Supplementary file 1 — Supplementary Material 1. [file 12887_2026_7010_MOESM1_ESM.pdf]

Self-Certification for Determining Whether Human Subjects Are Involved In Research When Obtaining Coded Private Information (Data) and/or Biological Specimens

University of California, San Francisco (UCSF)
Committee on Human Research (CHR)

Instructions:

1. Use this form if you need to provide funding agencies, administrators or collaborators with documentation that your research project does not require IRB review at UCSF. Keep a copy of the form in the PI's research file. Do **not** submit a copy to the CHR.

2. For help making this determination, review the Determining Whether Human Subjects Are Involved in Research Decision Tree and the Non Human Subjects Research guidance page. Contact the CHR at 415-476-1814 or email chr@ucsf.edu if you have questions.

3. Do **not** use this form for human stem cell research, which requires review by the GESCR Committee and may require IRB review.

**Principal Investigator:**		
Name and Degree<br>Timur Durrani, MD	Institution<br>UCSF	Department<br>Medicine
Mailing Address<br>Box 1369, UCSF	Phone Number<br>415-206-6581	E-mail Address<br>timur.durrani@ucsf.edu

Study/Grant Title/Award No.:

If your research meets the following conditions, the use of de-identified or coded private information (data) and/or biological specimens does not meet the definition of a *human subject* and does not require IRB/CHR review at UCSF:

1. The research is not regulated by the Food and Drug Administration (FDA).

   AND

2. No one on the UCSF research team has access to identifiable information and one or more of the following apply (check all applicable boxes):

   - [x] The researcher(s) receive de-identified data or specimens.
   - [ ] The researcher(s) receive coded data or specimens AND one or more of the following apply:
     - [ ] The key to decipher the code is destroyed before the research begins.
     - [ ] The PI and holder of the key enter into an agreement prohibiting the release of the key under any circumstances.
     - [ ] There are IRB-approved written policies for the repository or data management that prohibit the release of the key.
     - [ ] There are other legal requirements prohibiting the release of the key under any circumstances.

Principal Investigator's Certification:

I certify that the information provided in this application is complete and correct.

[signature]	11/14/2023
**Principal Investigators Signature**	**Date**
