# Supplementary material for: Trends in pediatric household cleaning product exposures before and during the COVID-19 pandemic: a national poison data system analysis (2016–2023)
Source: BMC Pediatr. 2026 May 21;26:658. doi: 10.1186/s12887-026-07010-2 (PMC13371303; doi:10.1186/s12887-026-07010-2)
Supplement: Supplementary file 2 — Supplementary Material 2: Supplemental Table 1. APC Cleaning Substances (Household) Generic Code Name and Number. Supplemental Figure 1. Flow diagram of pediatric household cleaning product exposures included in the analysis. Starting with 640,410 unique reported exposures, records with missing, blank, or “unknown” values in key variables (Generic Code, Age, Age Unit, Gender, Exposure Site, Caller Site, Management Site, or Medical Outcome) were sequentially excluded, leaving 633,317 exposures for the main analyses. Subsequent analyses by single product category excluded exposures involving multiple products, resulting in 614,097 exposures included in these comparisons. Supplemental Table 2. Household cleaning product exposure prevalence by year for all pediatric exposures, NPDS, 2016–2023 (n = 633,317). Supplemental Table 3. Medical outcomes of pediatric household cleaning product exposures stratified by single- and multiple-substance exposures, 2016–2023. [file 12887_2026_7010_MOESM2_ESM.docx]

**Supplemental Materials**

**Supplemental Table 1.** APC CLEANING SUBSTANCES (HOUSEHOLD) Generic Code Name and Number

| **Household Cleaning Product Category** | **Generic Code Name** | **Generic Code Number** |
| --- | --- | --- |
| **Acids** |  |  |
|  | Drain cleaners acid | 0115287 |
|  | Drain cleaners hydrochloric acid | 0201005 |
|  | Drain cleaners sulfuric acid | 0201006 |
|  | Misc cleaning agents acids | 0115282 |
|  | Oven cleaners acids | 0115283 |
|  | Rust removers acids other than hydrofluoric | 0115284 |
|  | Toilet bowl cleaners acids | 0115285 |
|  | Wall/floor/tile/all-purpose cleaning agents acids | 0115286 |
| **Alcohols glycols** |  |  |
|  | Glass cleaners isopropyl alcohol | 0025280 |
|  | Misc cleaning agents ethanol | 0019280 |
|  | Misc cleaning agents glycols | 0051280 |
|  | Misc cleaning agents isopropyl alcohol | 0025281 |
|  | Misc cleaning agents methanol | 0031280 |
|  | Spot remover/dry cleaning agent glycols | 0051281 |
|  | Spot remover/dry cleaning agent isopropyl alcohol | 0025282 |
|  | Wall/floor/tile/all-purpose cleaning agents ethanol | 0019281 |
|  | Wall/floor/tile/all-purpose cleaning agents glycols | 0051282 |
|  | Wall/floor/tile/all-purpose cleaning agents isopropyl alcohol | 0025283 |
|  | Wall/floor/tile/all-purpose cleaning agents methanol | 0031281 |
| **Alkalis** |  |  |
|  | Drain cleaners: alkalis | 0011289 |
|  | Misc cleaning agents alkalis | 0011284 |
|  | Oven cleaners alkalis | 0011285 |
|  | Rust removers alkalis | 0011286 |
|  | Toilet bowl cleaners alkalis | 0011287 |
|  | Wall/floor/tile/all-purpose cleaning agents alkalis | 0011288 |
| **Ammonia** |  |  |
|  | Ammonia excluding cleaning agents – all are multiple ingredient | Multiple |
|  | Ammonia cleaners | 0173280 |
|  | Glass cleaners ammonia containing | 0173281 |
| **Bleach** |  |  |
|  | Bleaches: Hypochlorite (Liquid and Dry) | 0042280 |
|  | Disinfectants: Hypochlorite (Non-Bleach Products) | 0042281 |
|  | Bleaches: Other or Unknown (Household) | 0077282 |
|  | Bleaches: Non-Hypochlorite | 0077280 |
| **Borates (mostly bleaches)** |  |  |
|  | Bleaches borates | 0062280 |
| **Cationics** |  |  |
|  | Industrial cleaners cationics | 0014000 |
|  | Wall/floor/tile/all-purpose cleaning agents cationics | 0014283 |
| **Dishwasher (all are multi)** |  |  |
|  | Automatic dishwasher detergents granules | Multiple |
|  | Automatic dishwasher detergents liquids | Multiple |
|  | Automatic dishwasher rinse agents | Multiple |
|  | Other or unknown type of automatic dishwasher detergent | Multiple |
| **HF** |  |  |
|  | Hydrofluoric acid |  |
|  | Hydrofluoric acid or bifluoride wheel cleaners | 0201007 |
|  | Rust removers hydrofluoric acid | 0118280 |
| **Laundry (mostly spot removers)** |  |  |
|  | Enzyme and/or microbiological laundry additives | Multiple |
|  | Fabric softener/antistatic agents | Multiple |
|  | Laundry bluing and/or brightening agents | Multiple |
|  | Laundry detergent granules | Multiple |
|  | Laundry detergent liquids | Multiple |
|  | Laundry detergent other | Multiple |
|  | Laundry prewash stain removers aerosol | Multiple |
|  | Laundry prewash stain removers liquids | Multiple |
|  | Laundry prewash stain removers liquid surfactants | Multiple |
|  | Laundry prewash stain removers other unknown | Multiple |
|  | Other or unknown laundry additive or miscellaneous product | 0077313 |
|  | Spot remover/dry cleaning agent other hydrocarbon | 0039281 |
|  | Spot remover/dry cleaning agent other halogenated | 0039280 |
|  | Spot remover/dry cleaning agent perchlorethylene | 0170280 |
| **Phenol** |  |  |
|  | Disinfectants phenol | 0040280 |
|  | Misc cleaning agents phenol (excluding disinfectants) | 0040281 |
| **Pine oil** |  |  |
|  | Disinfectants pine oil | 0039282 |
| **Soaps** |  |  |
|  | Anionic or nonionic cleansers | 0013280 |
|  | Anionic or nonionic hand dishwashing detergents | Multiple |
|  | Bath oils and/or bubble baths | Multiple |
|  | Glass cleaners anionics or nonionics | 0013282 |
|  | Industrial cleaners anionics or nonionics | Multiple |
|  | Miscellaneous cleaning agents anionics or nonionics | 0013000 |
|  | Other or unknown type of household hand dishwashing detergent | Multiple |
|  | Oven cleaners detergent type | 0013289 |
|  | Soaps bar, hand, complexion | Multiple |
|  | Spot removers/dry cleaning agents anionics or nonionics | Multiple |
|  | Wall/floor/tile/all-purpose cleaning agents anionics or nonionics | 0013288 |
|  | Anionic or nonionic rust remover | 0013286 |
| **Starches** |  |  |
|  | Starches fabric finishes or sizing | 0036280 |
| **Unknown** |  |  |
|  | Carpet upholstery leather or vinyl cleaners | 0013290 |
|  | Disinfectants other unknown | 0077286 |
|  | Drain cleaners other unknown | 0077314 |
|  | Glass cleaners other unknown | 0077294 |
|  | Misc cleaning agents other unknown | 0077303 |
|  | Other or unknown types of household cleansers | 0077282 |
|  | Oven cleaners other unknown | 0077306 |
|  | Rust removers other unknown | 0077307 |
|  | Spot removers/dry cleaning agents other unknown | 0013287 |
|  | Toilet bowl cleaners other unknown | 0077309 |
|  | Wall/floor/tile/all-purpose cleaning agents other unknown | 0077311 |

**Supplemental Figure 1.** Flow diagram of pediatric household cleaning product exposures included in the analysis.


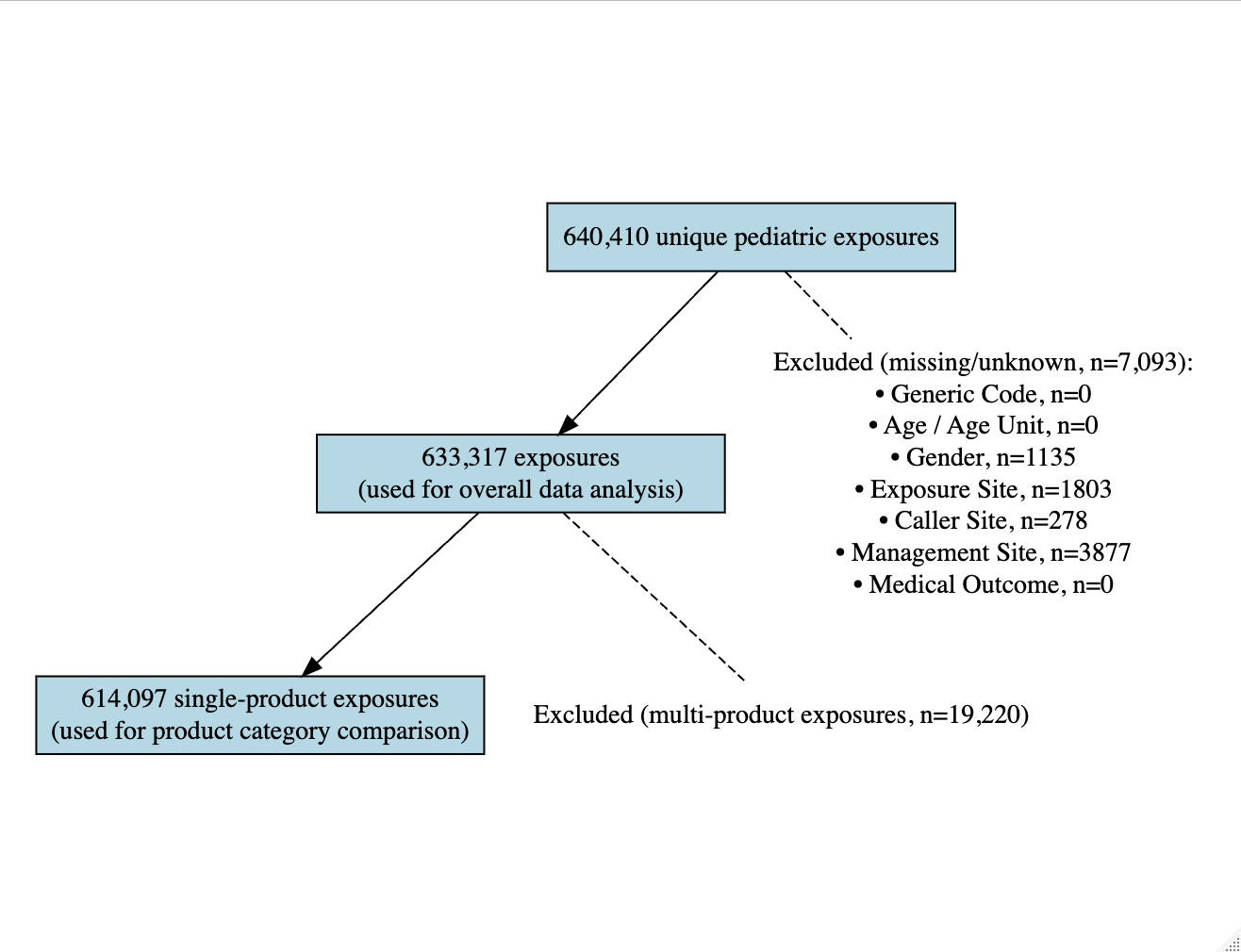
Starting with 640,410 unique reported exposures, records with missing, blank, or “unknown” values in key variables (Generic Code, Age, Age Unit, Gender, Exposure Site, Caller Site, Management Site, or Medical Outcome) were sequentially excluded, leaving 633,317 exposures for the main analyses. Subsequent analyses by single product category excluded exposures involving multiple products, resulting in 614,097 exposures included in these comparisons.

**Supplemental Table 2.** Household cleaning product exposure prevalence by year for all pediatric exposures, NPDS, 2016–2023 (n = 633,317)

| \| **Cleaning Product Category** \| **2016** \| **2017** \| **2018** \| **2019** \| **2020** \| **2021** \| **2022** \| **2023** \| **Total** \| \| --- \| --- \| --- \| --- \| --- \| --- \| --- \| --- \| --- \| --- \| \| **Acid** \| 2957 \| 2818 \| 2993 \| 2655 \| 2709 \| 2492 \| 2744 \| 2964 \| 22332 \| \| **Alcohols/Glycols** \| 3708 \| 3796 \| 2781 \| 2270 \| 2044 \| 1762 \| 1619 \| 1585 \| 19565 \| \| **Alkali** \| 11960 \| 11397 \| 10577 \| 9535 \| 8965 \| 7852 \| 8009 \| 7871 \| 76166 \| \| **Ammonia** \| 1239 \| 1130 \| 1050 \| 861 \| 599 \| 465 \| 383 \| 377 \| 6104 \| \| **Bleach** \| 14560 \| 13517 \| 11682 \| 11695 \| 13190 \| 11308 \| 9667 \| 9250 \| 94869 \| \| **Borates** \| 102 \| 187 \| 828 \| 543 \| 123 \| 128 \| 120 \| 56 \| 2087 \| \| **Cationic** \| 2703 \| 2877 \| 2527 \| 2316 \| 2648 \| 2483 \| 2380 \| 2,517 \| 20451 \| \| **Hydrofluoric Acid** \| 38 \| 39 \| 32 \| 23 \| 26 \| 22 \| 14 \| 17 \| 211 \| \| **Laundry** \| 13853 \| 11891 \| 11631 \| 11157 \| 10466 \| 10103 \| 9432 \| 9429 \| 87962 \| \| **Dishwasher** \| 8210 \| 7704 \| 7204 \| 7391 \| 7125 \| 6269 \| 5814 \| 5767 \| 55484 \| \| **Phenol** \| 450 \| 353 \| 215 \| 150 \| 144 \| 133 \| 145 \| 141 \| 1731 \| \| **Pine Oil** \| 1828 \| 1607 \| 1446 \| 1285 \| 1486 \| 1523 \| 1677 \| 812 \| 11664 \| \| **Soap** \| 13597 \| 12689 \| 12412 \| 11729 \| 12679 \| 11261 \| 10361 \| 10422 \| 95150 \| \| **Starches** \| 166 \| 166 \| 136 \| 108 \| 69 \| 40 \| 59 \| 39 \| 783 \| \| **Unknown** \| 15315 \| 14575 \| 14579 \| 14645 \| 16121 \| 14966 \| 14783 \| 14390 \| 119374 \| \| **Multiple** \| 2422 \| 2506 \| 2366 \| 2201 \| 2875 \| 2233 \| 2243 \| 2374 \| 19220 \| \| **Other** \| 25 \| 26 \| 23 \| 19 \| 13 \| 18 \| 25 \| 15 \| 164 \| \| **Total** \| 93133 \| 87278 \| 82482 \| 78583 \| 81282 \| 73058 \| 69475 \| 68026 \| 633317 \| |
| --- | --- | --- | --- | --- | --- | --- | --- | --- | --- | --- | --- | --- | --- | --- | --- | --- | --- | --- | --- | --- | --- | --- | --- | --- | --- | --- | --- | --- | --- | --- | --- | --- | --- | --- | --- | --- | --- | --- | --- | --- | --- | --- | --- | --- | --- | --- | --- | --- | --- | --- | --- | --- | --- | --- | --- | --- | --- | --- | --- | --- | --- | --- | --- | --- | --- | --- | --- | --- | --- | --- | --- | --- | --- | --- | --- | --- | --- | --- | --- | --- | --- | --- | --- | --- | --- | --- | --- | --- | --- | --- | --- | --- | --- | --- | --- | --- | --- | --- | --- | --- | --- | --- | --- | --- | --- | --- | --- | --- | --- | --- | --- | --- | --- | --- | --- | --- | --- | --- | --- | --- | --- | --- | --- | --- | --- | --- | --- | --- | --- | --- | --- | --- | --- | --- | --- | --- | --- | --- | --- | --- | --- | --- | --- | --- | --- | --- | --- | --- | --- | --- | --- | --- | --- | --- | --- | --- | --- | --- | --- | --- | --- | --- | --- | --- | --- | --- | --- | --- | --- | --- | --- | --- | --- | --- | --- | --- | --- | --- | --- | --- | --- | --- | --- | --- | --- | --- | --- | --- | --- | --- |

Annual counts of pediatric exposures for each household cleaning product category from 2016 through 2023.

**Supplemental Table 3.** Medical outcomes of pediatric household cleaning product exposures stratified by single- and multiple-substance exposures, 2016–2023.

| \| **Medical Outcome** \| **Single substance exposures, n (%)** \| **Multiple substance exposures, n (%)** \| \| --- \| --- \| --- \| \| **Death** \| 5 (<0.1%) \| 1 (<0.1%) \| \| **Major** \| 334 (0.1%) \| 50 (0.6%) \| \| **Moderate** \| 5919 (2.6%) \| 413 (4.9%) \| \| **Minor** \| 89285 (38.6%) \| 3546 (42.4%) \| \| **No Effect** \| 135300 (58.5%) \| 4342 (51.9%) \| \| **Confirmed non-exposure** \| 268 (0.1%) \| 13 (0.2%) \| \| **Total** \| 231111 \| 8365 \| |
| --- | --- | --- | --- | --- | --- | --- | --- | --- | --- | --- | --- | --- | --- | --- | --- | --- | --- | --- | --- | --- | --- | --- | --- | --- |

Medical outcomes of pediatric household cleaning product exposures, stratified by single- and multiple-substance exposures (2016–2023). Analyses are restricted to cases with known medical outcomes; percentages are calculated within each exposure group.
